# Distinct cellular responses to replication stress leading to apoptosis or senescence

**DOI:** 10.1002/2211-5463.12632

**Published:** 2019-04-13

**Authors:** Emilie Lukášová, Martina Řezáčová, Alena Bačíková, Ludmila Šebejová, Jiřina Vávrová, Stanislav Kozubek

**Affiliations:** ^1^ Department of Cell Biology and Radiobiology Institute of Biophysics The Czech Academy of Sciences Brno Czech Republic; ^2^ Department of Medical Biochemistry Faculty of Medicine in Hradec Králové Charles University in Prague Hradec Králové Czech Republic; ^3^ Department of Internal Medicine – Hematology and Oncology University Hospital Brno and Faculty of Medicine Masaryk University Brno Czech Republic; ^4^ Department of Radiobiology Faculty of Military Health Sciences Hradec Králové University of Defence Brno Hradec Králové Czech Republic

**Keywords:** apoptosis, ATR inhibitor, Chk1 inhibitor, lamin B receptor, replication stress, senescence

## Abstract

Replication stress (RS) is a major driver of genomic instability and tumorigenesis. Here, we investigated whether RS induced by the nucleotide analog fludarabine and specific kinase inhibitors [e.g. targeting checkpoint kinase 1 (Chk1) or ataxia telangiectasia and Rad3‐related (ATR)] led to apoptosis or senescence in four cancer cell lines differing in *TP53* mutation status and expression of lamin A/C (LA/C). RS resulted in uneven chromatin condensation in all cell types, as evidenced by the presence of metaphasic chromosomes with unrepaired DNA damage, as well as detection of less condensed chromatin in the same nucleus, frequent ultrafine anaphase bridges, and micronuclei. We observed that responses to these chromatin changes may be distinct in individual cell types, suggesting that expression of lamin A/C and lamin B1 (LB1) may play an important role in the transition of damaged cells to senescence. MCF7 mammary carcinoma cells harboring wild‐type p53 (WT‐p53) and LA/C responded to RS by transition to senescence with a significant reduction of lamin B receptor and LB1 proteins. In contrast, a lymphoid cancer cell line WSU‐NHL (WT‐p53) lacking LA/C and expressing low levels of LB1 died after several hours, while lines MEC‐1 and SU‐DHL‐4, both with mutated p53, and SU‐DHL‐4 with mutations in LA/C, died at different rates by apoptosis. Our results show that, in addition to being influenced by p53 mutation status, the response to RS (apoptosis or senescence) may also be influenced by lamin A/C and LB1 status.

**Abbreviations**
ATM: ataxia telangiectasia mutated
ATR: ataxia telangiectasia and Rad3‐related
ATRi: ATR inhibitor
Chk1: checkpoint kinase 1
Chk1i: Chk1 inhibitor
DDR: DNA damage response
DNA‐PK: DNA‐dependent protein kinase
DNA‐PKi: DNA‐PK inhibitor
DSB: DNA double‐strand breaks
FLU: fludarabine
INM: inner nuclear membrane
LA/C: lamin A/C
LB1: lamin B1
LBR: lamin B receptor
RPA: replication protein A
RS: replication stress
RT: room temperature
SA‐β‐gal: senescence‐associated β‐galactosidase
ssDNA: single‐stranded DNA
WT: wild‐type

Conditions that interfere with DNA replication and lead to fork stalling or slowing are collectively referred to as DNA replication stress (RS). RS is a major driver of genomic instability and tumorigenesis 1. The main causes of endogenous DNA damage in proliferating cells are errors occurring during DNA replication 1, 2, 3. RS induces a specialized branch of DNA damage response (DDR); the S‐phase checkpoint, initiated by the generation of single‐stranded DNA stretches at stalled or damaged forks triggering the activation of the kinase ataxia telangiectasia and Rad‐3 related (ATR) and checkpoint kinase 1 (Chk1) 4, 5, 6, 7. This pathway is fundamental to delaying cell cycle progression to give the cell time to repair DNA damage as much as possible, recover stalled replication forks and complete replication before entry into mitosis. However, certain regions in the genome, including common fragile sites, are difficult to replicate and can persist in an under‐replicated state during cell progression into mitosis. This frequent phenomenon may be a fundamental aspect of cell division 3, 8, 9, 10, 11 and can be artificially intensified by the use of specific chemotherapeutics that by reducing the nucleotide pool or inhibiting DNA polymerase affect DNA replication. Chk1 or ATR inhibition has been extensively explored as a chemotherapy potentiating strategy for the treatment of cancer 12, 13.

Chk1 is specifically phosphorylated by ATR 4, 5 while Chk2 is a specific substrate of the kinase ataxia telangiectasia mutated (ATM) 1, 14, 15. In addition to their specificity for particular checkpoint kinases, another key difference between ATM and ATR is their activation in response to genotoxic stress. ATM is activated exclusively by DNA double‐strand breaks (DSBs), while ATR is activated by the presence of replication protein A (RPA)‐coated by single‐stranded DNA (ssDNA) regions 16. These regions are generated by the uncoupling of replicative helicase in areas of replication fork stalling or by DSB end resection during homologous recombination 2, 16. In addition, whereas DSBs activate ATM at any stage of the cell cycle, the response of ATR to ssDNA is restricted to the S and G2 phases 17, 18. If the DNA damage persists for a longer time, the cell cycle arrest may become permanent, navigating cells to an irreversible state of quiescence referred to as cellular senescence 16. Cellular senescence induced in response to diverse stress conditions 19, 20, 21, 22, 23, 24 is accompanied by a set of characteristic morphological and physiological features that distinguish senescent cells from those that are proliferative, quiescent or differentiated. These senescence‐associated markers typically include irreversible proliferation arrest, enlarged cellular morphology, senescence‐associated β‐galactosidase (SA‐β‐gal) expression, increased content of nuclear heterochromatin, changes in gene transcription and senescence‐associated secretory phenotype 24. As already stated, an important functional marker of senescence is the phosphorylation of H2AX (γH2AX) in reaction to DDR. We recently found that genotoxic stress induced by γ‐irradiation results in cellular senescence in MCF7 and U2OS cancer cell lines, produced senescence‐associated markers, and caused the downregulation of the lamin B receptor (LBR) and consequentially the protein lamin B1 (LB1) 25. LB1 loss in senescence has been previously observed by several authors; however, the reason for this loss was not elucidated 26, 27, 28, 29, 30. Chandra *et al*. 29 and independently Sadaie *et al*. 28 reported that despite the global reduction of the LB1 protein level, LB1 binding increased in a small subset of gene‐rich regions with histone H3K27me3, implying that the loss of LB1 might be associated with architectural changes of chromatin. In our functional study where LBR was silenced by shRNA, we discovered concomitant reduction of LBR and LB1 levels suggesting interrelated regulation of both proteins 25. Our results also showed that the reduced expression of LBR resulted in the relocation of centromeric heterochromatin from the inner nuclear membrane (INM) to the nucleoplasm and its unfolding. This phenotype was observed not only in senescent cells but also in cells treated with LBR‐specific shRNA, indicating that LBR tethers heterochromatin to INM in cancer cells and LB1 is an integral part of this anchoring. We propose that down‐regulation of LBR and LB1 at the beginning of senescence is necessary for the release of heterochromatin from binding to lamina to achieve changes in chromatin structure and gene expression leading to the cessation of cell proliferation.

In this work, we analyzed the effect of RS on the induction of apoptosis or senescence in cancer cells. For induction of the RS, we used a purine analog fludarabine (FLU), which inhibits DNA synthesis by interfering with ribonucleotide reductase and DNA polymerase 31.

To have a greater effect on replication, we also applied specific small molecule inhibitors of Chk1 (SCH900776) 32, 33 and ATR (VE821) 34, 35. We were interested in whether the ssDNA induced at stalled replication foci by this treatment would result in cell death or the transition of the cells to senescence and whether this senescence would be accompanied by the loss of LBR and LB1 that we observed after γ‐irradiation of cancer cells 25. The study was performed using three lymphoid cancer cell lines and one line of mammary carcinoma. These cells differed in mutations specific for tumor transformation as well as in the mutation status of p53 and lamin A/C (Table 1).

**Table 1:** Cell line properties

Cell line properties	Cell line			
	WSU‐NHL	SU‐DHL‐4	MEC‐1	MCF7
p53 status	Wild‐type	Mutation	Mutation	Wild‐type
Lamin A/C status	Lack	Mutation	Normal	Normal
Lamin B1 status	Lack	Normal	Normal	Normal

## Materials and methods

### Cell lines and culture

MCF7, breast adenocarcinoma cells (ATCC, Manassas, VA, USA) were grown in Dulbecco's modified Eagle's medium with 10% fetal bovine serum (Biomedicals, Wien, Austria), 100 U·mL^−1^ penicillin and 0.1 mg·mL^−1^ streptomycin (Sigma‐Aldrich, MERCK KGaA, Darmstadt, Germany). Cell lines representing B‐cell malignancies, SU‐DHL‐4 (diffuse large B‐cell lymphoma), WSU‐NHL (diffuse large B‐cell lymphoma) and MEC‐1 (chronic lymphocytic leukemia in prolymphocytoid transformation), were obtained from the Leibnitz Institute DSMZ‐German Collection of Microorganisms and Cell Cultures. These cells were cultured in Iscove's modified Dulbecco's medium from Sigma‐Aldrich supplemented with fetal bovine serum (MP Biomedicals, Wien, Austria). All cells were cultured in a humidified atmosphere at 37 °C and 5% CO_2_. The *TP53* mutation status in cell lines from B‐cell malignancies was verified by yeast functional analysis (FASAY) coupled to sequencing 36.

### Drugs

Fludarabine was purchased from Sigma‐Aldrich. Chk1 inhibitor SCH900776 (Merck, MWRCK KGaA, Darmstadt, Germany; MK‐8776) was kindly provided by K. Paruch (Department of Chemistry, Masaryk University). The inhibitor was dissolved as 100 μm stock solution and stored at room temperature (RT). Before use it was diluted in culture medium to 200 nm. A selective ATR inhibitor, VE‐821, was purchased from APIs Chemical Co., Ltd, Shanghai, China, KU55933, the ATM inhibitor, was from Tocris Bioscience (Ellisville, MO, USA) and NU7441 and the DNA‐dependent protein kinase inhibitor (DNA‐PKi) were from Axon Medchem (Groningen, the Netherlands). The inhibitors were dissolved in dimethyl sulfoxide as 10 mm aliquots and stored at −80 °C. The desired final concentrations were achieved by dilution with culture medium. The final concentrations were 10 μm for VE821 and KU55933, and 1 μm for NU7441.

### Induction of replication stress

Twenty‐four hours after cell seeding, FLU was added at a concentration 5 or 10 μg·mL^−1^, and cells were incubated at 37 °C for 2 h before addition of the inhibitors. Cells were then incubated for 3, 6, 14, 24 or 48 h. After treatment the cells were washed, supplied with fresh medium and incubated for a range of time intervals before processing. The cells, incubated with different inhibitors for different times, are marked in figure legends thus: ‘F10+Sch 48/72’ indicates incubation with 10 μg·mL^−1^ fludarabine + 200 nm Sch900776 for 48 h followed by incubation in fresh medium for an additional 72 h.

### Antibodies and immunofluorescence

MCF7 cells cultured on microscope slides were withdrawn at different time intervals after exposure to RS and washed twice in PBS before fixation. Cells growing in suspension were harvested by centrifugation at selected time intervals after exposure to RS, washed with PBS and seeded on slides where they were allowed to attach for 5 min at RT. The slides were then immersed into 4% paraformaldehyde for cell fixation for 10 min at 21 °C, rinsed quickly in PBS, washed three times for 5 min in PBS, permeabilized in 0.2% Triton X‐100/PBS for 15 min at RT and washed twice for 5 min. Prior to incubation with primary antibodies (overnight at 4 °C), the cells were blocked with 5% inactivated fetal calf serum + 2% bovine serum albumin/PBS for 30 min at RT. Antibodies from two different hosts (rabbit and mouse) were used on each slide to detect two different antigens in the same nuclei. Anti‐H2AX phosphorylated at serine 139 (no. 05‐636), anti‐H3K9Me3 (no. 05‐1242), anti‐HP1γ (no. MAB3450), anti‐p21 (no. 05‐345), and anti‐p16 (no. MAB4133) antibodies were from Millipore, Guyancourt, Francie; anti‐53BP1 (no. 4937), anti‐p53 (no. 2524T), anti‐phospho‐p53‐ser15 (no. 9286), and anti‐β‐actin (no. 4970) antibodies were from Cell Signaling Technology, Leiden, Netherland; anti‐active‐Caspase‐3 (no. ab32042); anti‐LB1 (no. ab8982), anti‐LBR (no. ab32535) and anti‐emerin (no. ab54996) antibodies were from Abcam, Cambridge, UK. Anti‐lamin A/C (3SAB42000236) was from Sigma‐Aldrich. The secondary antibodies were affinity purified‐FITC conjugated donkey anti‐mouse and affinity purified Cy3‐conjugated donkey anti‐rabbit from Jackson ImmunoResearch Laboratories (West Grove, PA, USA). Cells were preincubated with 5% donkey serum/PBS for 30 min at RT and then incubated with a mixture of both antibodies on each slide for 1 h in the dark at RT. This was followed by washing (three times for 5 min each) in PBS. Cells were counterstained with 1 μm TO‐PRO‐3 (Molecular Probes, Eugene, OR, USA) in 2× saline sodium citrate (SSC) prepared fresh from a stock solution. After brief washing in 2× SSC, Vectashield medium (Vector Laboratories, Burlingame, CA, USA) was applied for final mounting.

### Confocal fluorescence microscopy

The immunofluorescence images were obtained with a high‐resolution Leica DM RXA confocal cytometer (Leica, Wetzlar, Germany), equipped with an oil immersion Plan Fluotar objective (×100/NA 1.3) and a CSU 10a Nipkow disc (Yokogawa, Japan) for confocal imaging. A CoolSnap HQ CCD‐camera (Photometrix, Tucson, AZ, USA) and an Ar/Kr laser (Innova 70C Spectrum; Coherent, Santa Clara, CA, USA) were used for image acquisition. Automated exposure, image quality control, image analysis and other procedures were performed using acquiarium software 37. The exposure time and dynamic range of the camera in the red, green and blue channels were adjusted to the same values for all slides to obtain quantitatively comparable images. Forty serial optical sections were captured at 0.2 μm intervals (along the *z*‐axis). A total of 100–300 cells were recorded for each set of conditions and experiments were repeated in duplicate or triplicate. Results are reported as SEM. Student's *t‐*test was used for statistical comparison of specific points. DSBs were detected by antibodies to γH2AX colocalizing with anti‐53BP1, single‐stranded DNA induced by Chk1 inhibitor (Chk1i) or ATR inhibitor (ATRi) was detected by γH2AX antibody.

### Flow cytometry analysis

#### Detection of apoptosis

The Muse Annexin V (Sigma‐Aldrich) and Dead Cell Kit was used for the detection of early and late apoptosis (cells with permeable plasmatic membrane) on a Muse Cell Analyzer (Merck‐Millipore) according to the manufacturer's instructions. Cells were diluted between 1 × 10^5^ and 5 × 10^5^ mL^−1^. The results were stored in a data file. The summarized data show the cell concentration for the events in each quadrant and the percentage of gated cells in each quadrant as well as the concentration and percentage of total apoptotic cells. The data from the four independent experiments were used for the construction of bar graphs depicting the percentages of early and late apoptotic cells after different cell treatments.

#### Cell cycle evaluation

The cells were collected, washed with cold PBS and fixed with 70% ethanol. For the detection of low‐molecular‐mass fragments of DNA, cells were incubated for 5 min at RT in phosphate buffer (pH 7.8) and stained with propidium iodide in Vindelov's solution for 60 min at 37 °C in the dark. The DNA contents were determined by a CyAn DakoCytomation FACS analyzer (Beckman Coulter, Wien, Austria) immediately after incubation. At least 50 000 cells were analyzed per sample. List mode data were analyzed using summit V 4.3 software (Beckman Coulter). The data from at least three measurements for each treatment were used to construct histograms.

### WST test for cell viability

Cell viability assays were performed using Cell Proliferation Reagent WST‐1 (Roche Diagnostics GmbH, Wien, Austria) according to the manufacturer's procedures after 24, 48 and 72 h incubation. Each treatment was performed in five wells in a 96‐well plate. The results were detected at 450 nm on a Tecan Infinite 200 PRO multifunctional microplate reader (Tecan Austria GmbH, Grödig, Austria). The results represent an average from three independent experiments and are expressed with SEM.

### Senescence‐associated β‐galactosidase assay

Detection of SA**‐**β‐gal activity was performed by the method described by Dimri *et al*. 38 using the Senescence Detection Kit no. K320‐250 from Bio‐Vision Inc. (San Jose, CA, USA) following the manufacturer's instructions. Images were captured by an Olympus BX51 (Tokyo, Japan) microscope equipped with an Olympus DP72 camera and quick photo micro 2.3 software at magnification ×200. From each sample from the two independent experiments 100–200 cells were blind counted for SA‐β‐gal positivity. The recorded images were magnified in adobe photoshop for examination.

### SDS/PAGE and western blotting

Cells were washed in PBS, scraped in the presence of Complete Mini EDTA‐free protease inhibitors (Roche Diagnostics, Vienna, Austria; no. 04693159001) and PhosSTOP (a mixture of phosphatase inhibitors, Roche Diagnostics, no. 04906845001), and centrifuged. Cells were transferred into Laemmli SDS lysis buffer (50 mm Tris, pH 6.8; 100 mm DTT; 2% SDS; 0.1% bromophenol blue; 10% glycerol) supplemented with protease and phosphatase inhibitor cocktails, briefly sonicated and centrifuged at 14 000 ***g*** for 10 min. Protein concentration was measured by the Bradford assay (Bio‐Rad Laboratories Inc., Atlanta, GA, USA). Next, 0.01% of bromophenol blue and 100 mm DTT were added to lysates before separation on polyacrylamide gels. Twenty microgram of total proteins for each sample were separated by 8% SDS/PAGE (for LB1 and LBR) or by 15% SDS/PAGE (the other proteins). After electrophoresis, the proteins were transferred to PVDF membranes (Bio‐Rad) using semidry transfer. Staining of proteins with monoclonal antibodies was performed overnight. Detection was performed using SuperSignal West Pico Chemiluminescent Substrate Kits: no. 3482 Mouse IgG and no. 34083 Rabbit IgG detection kits (Thermo Scientific, Waltham, MA, USA). Β‐actin was used as a marker of equal protein loading. The protein signals were captured using a Fujifilm LAS‐3000 Imager (Stamford, CT, USA).

## Results

### Changes in chromatin structure induced by replication stress

**Figure 1:**
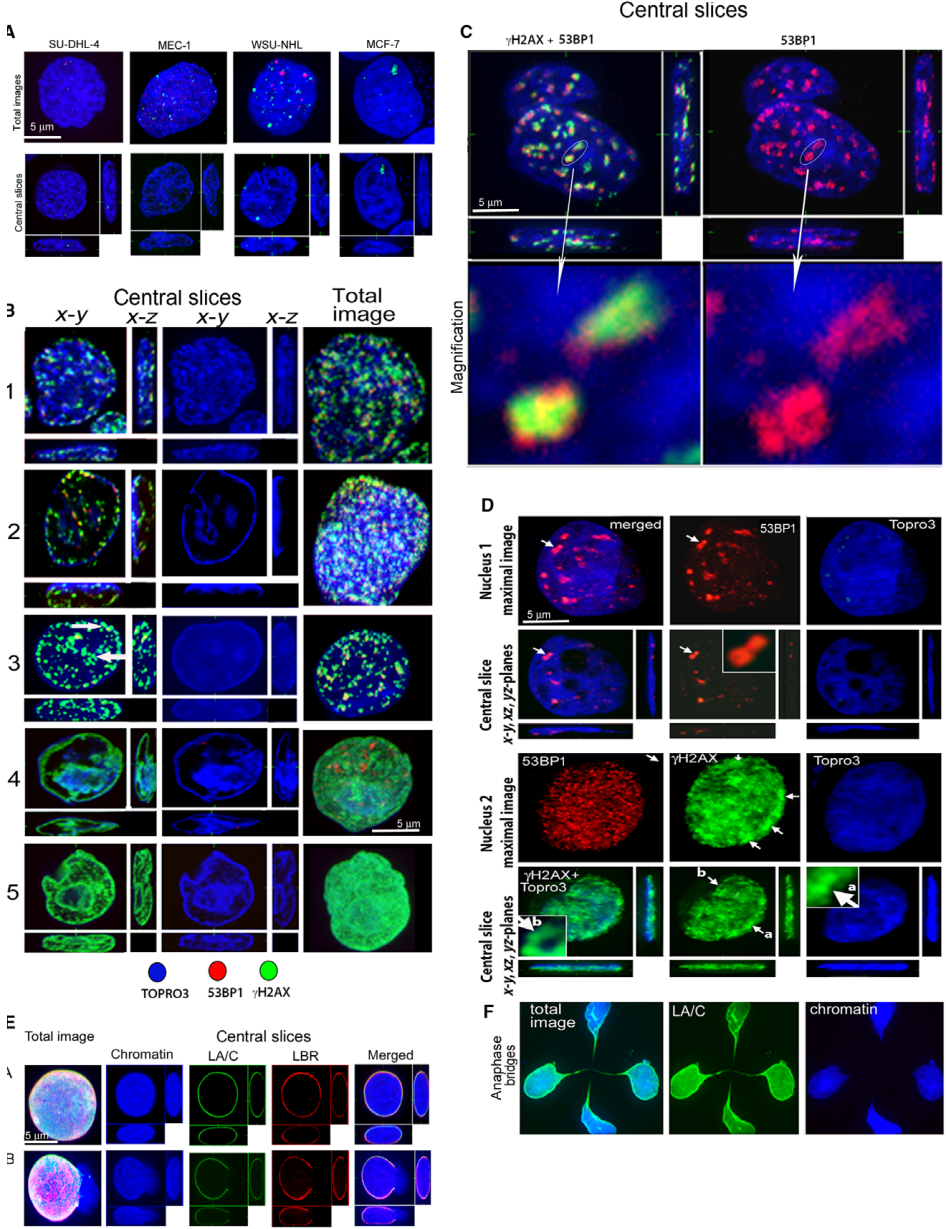
Changes in chromatin structure induced by FLU and Chk1i in tested cell lines. (A) Chromatin structure in control cells. Small red (53BP1) and green (γH2AX) foci represent DNA damage. (B) Different types of chromatin structure in SU‐DHL‐4 cells after treatment with FLU and Chk1i. (1) Exposure to FLU 5 μg·mL^−1^ (F5) for 24 h. (2) Exposure to F5 and Chk1i for 24 h. (3) Exposure to F5 and Chk1i for 48 h. Arrows show metaphasic chromosomes stained with γH2AX antibody. (4) Exposure to F5 for 24 h. (5) Exposure to F5 and Chk1i for 48 h. (C) Central slices through MEC‐1 cell exposed to F5 and Chk1i for 24 h. Chromatin (blue) condensed to metaphasic chromosomes is stained with γH2AX (green) and 53BP1 (red) antibodies. Arrows show magnification of metaphasic chromosomes stained with γH2AX and 53BP1 antibodies. (D) Chromatin structure of MCF7 cells exposed to FLU 10 μg·mL^−1^ (F10) and Chk1i for 48 h, subsequently released to a fresh medium and cultured for 7 days. Arrows show metaphasic chromosomes stained with 53BP1 (red) antibody at nucleus 1 and with γH2AX (green) antibody at nucleus 2. 53BP1 staining does not correspond to metaphasic chromosomes as does the γH2AX staining in this nucleus. MCF7 cells were treated with F10 because of their lower sensitivity to this nucleotide analog. (E) Inflated nuclei of MEC‐1 (A, B), emerging after exposure to F5 and Chk1i for 24 h. The nucleus B has a fissure in lamina resulting in release of chromatin into cytoplasm. (F) Anaphase bridges in MEC‐1 cells exposed to F5 for 24 h. Scale bars: 5 μm.

We initially evaluated the effects of RS on chromatin structure in lymphoid cancer cell lines SU‐DHL‐4, WSU‐NHL and MEC‐1, and in mammary carcinoma cell line MCF7. For this purpose, we used FLU and its combinations with Chk1i and ATRi. The majority of the chromatin of the control (untreated) lymphoid cells was condensed into irregularly distributed cords or globules that were densely stained by TO‐PRO‐3 and contained sporadic foci of phosphorylated H2AX (γH2AX) or 53BP1 (most pronounced in WSU‐NHL cells), marking DSB and/or ssDNA (Fig. 1A). The chromatin structure of MCF7 cells was distinct from lymphoid cell lines in terms of its lower condensation and globular structure. Exposure to RS induced by FLU or FLU with Chk1i increased the proportion of cells with DNA damage as seen from the presence of γH2AX and 53BP1 staining in all cell types and led to higher chromatin condensation (Fig. 1B,D). Chromatin changes were dependent on the length of exposure to FLU, its concentration (5 or 10 μg·mL^−1^) and also on parallel inhibition of Chk1 or Chk1 with ATM, or ATR. The five most common types of chromatin structure were identified in lymphoid cells. The results for SU‐DHL cells exposed to 5 μg·mL^−1^ of FLU for 14–48 h are depicted in Fig. 1B. These changes were observed in MEC‐1 and WSU‐NHL cells after just 3 h of exposure to FLU, indicating their more rapid response to this agent, but they were less frequent in MCF7 mammary carcinoma cells. The first type (Fig. 1B1) represents the overall globular chromatin structure in the whole volume of the nuclei probably reflecting the process of chromatin condensation. Antibodies for γH2AX and 53BP1 frequently reacted with these condensing chromosomes indicating a presence of ssDNA or possibly also common fragile sites. A peripheral heterochromatin layer contained condensing chromosomes arranged successively behind each other, with some of them being dispersed in the nucleus and distinguishable at the central slice through the nucleus. Various areas of condensing chromosomes were stained with antibodies to γH2AX, or 53BP1, or both these proteins concurrently. The structure of whole condensed metaphasic chromosomes can be distinguished, better than by TO‐PRO‐3, by staining of γH2AX and 53BP1, especially after image magnification (Fig. 1C). The second type of condensed chromatin (Fig. 1B2) is represented by a thick layer on the nuclear periphery and small heterochromatin islands inside the nuclei also containing condensing chromosomes stained with γH2AX and 53BP1 antibodies. The third type of chromatin (Fig. 1B3) is homogenously stained by TO‐PRO‐3 with no apparent chromatin condensation. The nucleus gives the impression of an inflated ball. Such nuclei frequently burst with chromatin protruding into the cytoplasm and were most frequent in the MEC‐1 cell line, representing about 10% of all cells (Fig. 1E). Large irregular spots of γH2AX, co‐localizing with smaller spots of 53BP1 were arranged successively behind each other in the peripheral heterochromatin layer and in chromatin with denser TO‐PRO‐3 staining in the nuclear space. The shape of some γH2AX spots resembles condensed metaphasic chromosomes (Fig. 1B3 arrows). The fourth and the fifth types of nuclei (Fig. 1B4,5) exhibited γH2AX expansion throughout the whole nuclear surface or even volume (we call it ‘pan‐nuclear γH2AX staining’). We hypothesize that pan‐nuclear γH2AX distribution mirrors a high level of ssDNA coated with phosphorylated H2AX. However, the presence of condensed metaphasic chromosomes together with incompletely condensed chromosomes in the nuclei of all these cells types does not correspond with the distribution of condensed metaphasic chromosomes in the pro‐metaphase of normal cells. Irregular condensation of chromatin into metaphasic chromosomes was also observed in MCF7 cells exposed to FLU and Chk1i for 48 h followed by incubation of the cells in fresh medium for 7 days (Fig. 1D). In comparison with lymphoid cells, chromatin in MCF7 cell lines was condensed more irregularly: there was a lower number of condensed metaphasic chromosomes as well as irregular condensed cords and chromatin of lower density (Fig. 1D). Condensed metaphasic chromosomes in these interphase nuclei were also stained by antibodies to γH2AX or 53BP1. Some nuclei contained condensed metaphasic chromosomes coated with γH2AX and only small 53BP1 foci densely scattered throughout the nucleus.

This unusual chromatin structure, which has previously not been described, was observed in all cell lines exposed to FLU or FLU accompanied with Chk1i and shows that it is characteristic for chromatin containing long and frequent stretches of ssDNA in cells exposed to RS.

**Figure 2:**
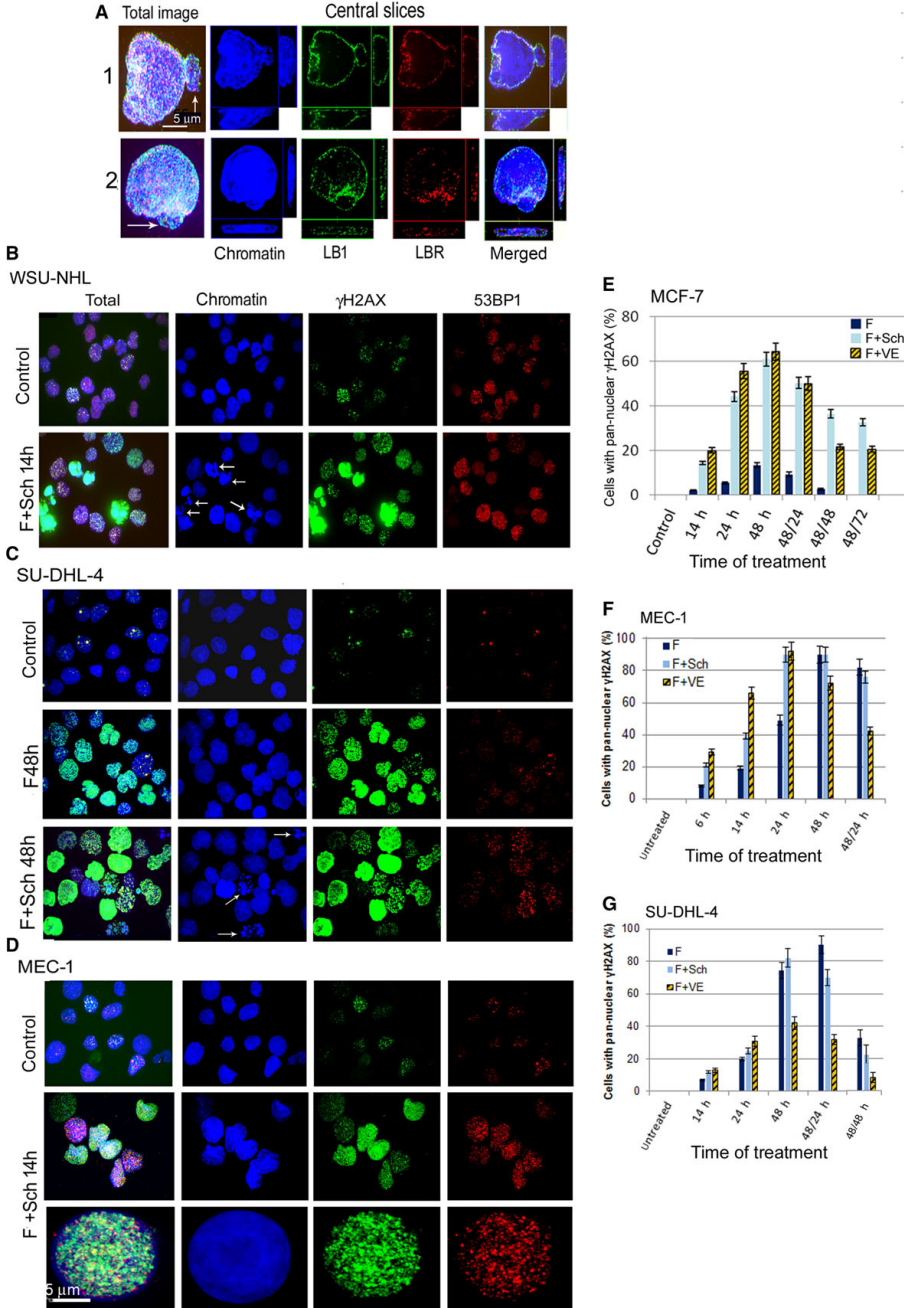
Pan‐nuclear γH2AX staining in the tested cell lines. (A) Formation of blebs and micronuclei (arrows) in SU‐DHL‐4 cells exposed to F5 with Chk1i for 24 h. (B–D) WSU‐NHL, SU‐DHL, MEC‐1 cells treated with F5 and Chk1i for different time intervals. (E–G) Proportions of MCF7, MEC‐1 and SU‐DHL‐4 cells with pan‐nuclear γH2AX staining after exposure to F5 with Chk1i or F5 with ATRi for individual time intervals. Example of the legend description: 48/24 indicates incubation with the RS inductors for 48 h, followed by a release of cells to fresh medium and culture for additional 24 h. Quantification of pan‐nuclear γH2AX staining is presented as the mean and SD from three independent experiments. The mean values were obtained by counting approximately 300 cells in each of the three independent experiments.

Exposure of all tested cell lines to FLU and Chk1i and/or ATRi also frequently resulted in the formation of ultrafine anaphase bridges (Fig. 1F) and was additionally characterized by the appearance of micronuclei formed by the release of nuclear protrusions (blebs) into the cytoplasm (Fig. 2A).

### Effect of Chk1 and ATR inhibition on pan‐nuclear γH2AX distribution in individual cell lines

Cells with pan‐nuclear γH2AX staining appeared after FLU treatment in all tested cell lines and their proportion increased after parallel treatment with Chk1i and/or ATRi (Figs 1B and 2B–D). In MCF7 and MEC‐1 cells, the addition of Chk1i or ATRi to FLU significantly increased the proportion of γ‐H2AX‐positive cells compared to FLU alone (Fig. 2E,F). However, in the case of SU‐DHL‐4 cells, we observed a rather inhibitory effect of ATRi on pan‐nuclear γ‐H2AX expansion (Fig. 2G). Cell fractions with pan‐nuclear γH2AX staining after FLU and FLU with Chk1i decreased after RS was stopped and the cells transferred to fresh medium and further culture. A slight decline was observed in MEC‐1 cells (Fig. 2F), whilst MCF7 and SU‐DHL‐4 cell lines demonstrated a much more pronounced change (Fig. 2E,G). The cell fractions with pan‐nuclear γH2AX staining decreased more rapidly after the addition of ATRi to FLU compared to Chk1i addition. This suggests that ATRi induced high RS that probably led to cell death, which was supported by the increased fraction of apoptotic cells after 24 h of exposure (Figs 4A and 5B,D).

**Figure 3:**
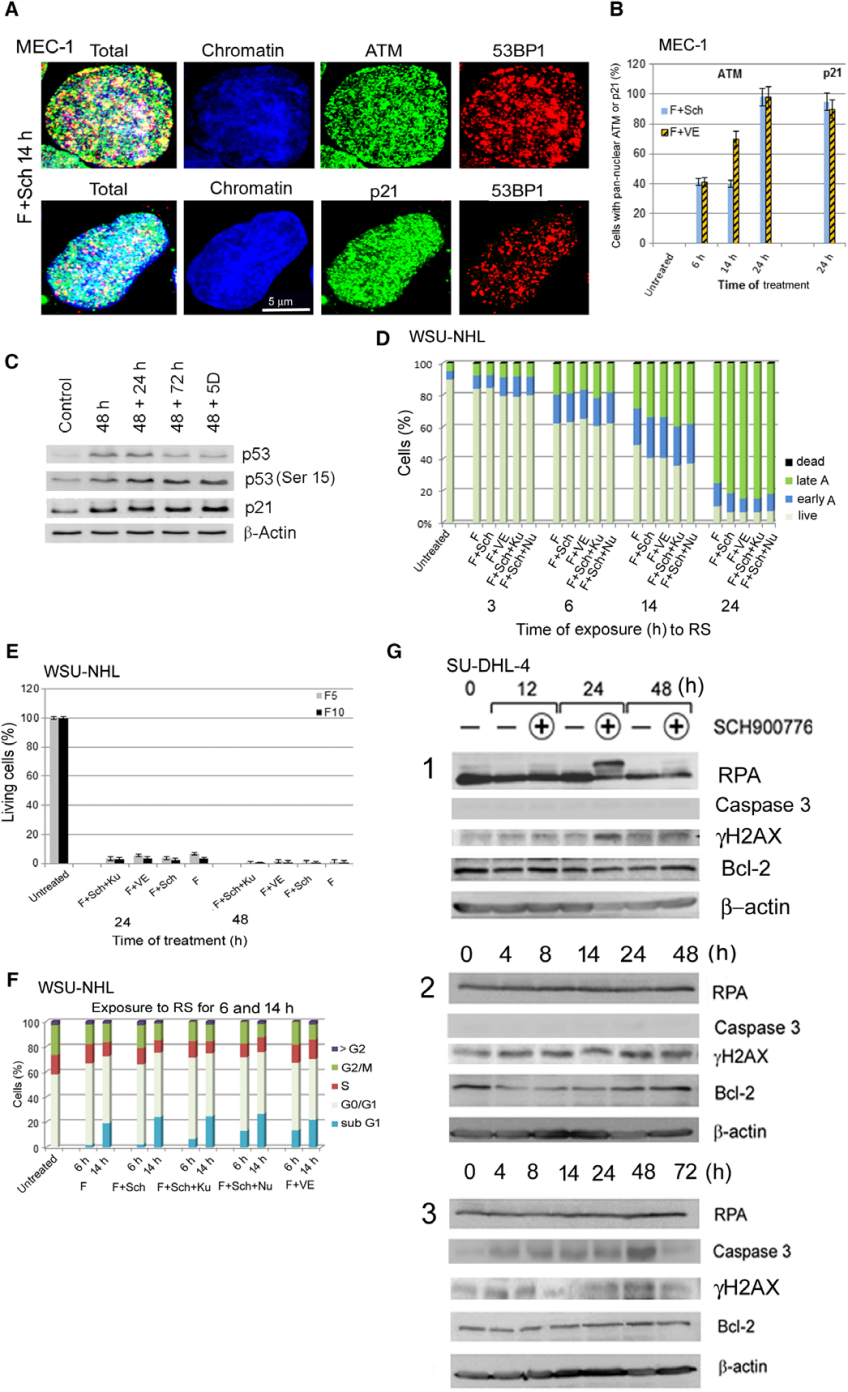
Impact of RS on selected proteins in MEC‐1 and SU‐DHL‐4 cells, and on the cell cycle profile and viability in WSU‐NHL cells. (A) Nuclei of MEC1 containing high level of ATM, p21 and 53BP1 proteins after treatment with FLU 5 μg·mL^−1^ (F5) and Chk1i (Sch) for 14 h. (B) Proportions of MEC‐1 cells containing pan‐nuclear staining of ATM and p21 proteins after treatment with F5 and Chk1i (Sch) or F5 and ATRi (VE). The mean values (with SD) were obtained by counting approximately 200 cells in two independent experiments. (C) Western blot of p53 and p21 proteins in MCF7 cells exposed to F10 + Chk1i for 48 h and after subsequent release of these cells to fresh medium and further culture. (D) Proportions of apoptotic WSU‐NHL cells after treatment with FLU 5 μg·mL^−1^ (F) in combination with Chk1i (F + Sch), or ATRi (F + VE), or with Chk1i and ATMi (F + Sch + Ku), or Chk1i and ATMi and DNA‐PKi (F + Sch + Nu) for different time. All results are presented as mean from three independent experiments. (E) Overall viability of WSU‐NHL cells exposed to the same stressors as in (C) for 24 and 48 h was detected by metabolic WST‐1 assay measuring overall cell viability. The results are presented as average from three independent experiments expressed with SEM (each experiment was performed in five different wells seeded with 5 × 10^4^ cells). (F) Changes in the cell cycle profile in WSU‐DHL cells exposed to FLU 5 μg·mL^−1^ (F), FLU with Chk1i (F + Sch), FLU with Chk1i and ATMi (F + Sch + Ku), and FLU with Chk1i and DNA‐PKi (F + Sch + Nu) for 6 or 14 h. The results are presented as mean from three independent experiments. (G1) Changes in the level of selected proteins in SU‐DHL cells exposed to FLU 5 μg·mL^−1^ or to FLU + Chk1i (Sch900776) for 12, 24 and 48 h. (G2) Changes of these proteins in cells exposed to FLU 5 μg·mL^−1^ for 48 h, released to fresh medium and cultured for additional 4–48 h. (G3) Changes in these proteins in cells exposed to FLU 5 μg·mL^−1^ + Chk1i (Sch900776) for 48 h, released to fresh medium and cultured for additional 4–72 h. Scale bar: 5 μm.

Exposure of MEC‐1 and SU‐DHL‐4 cells to FLU alone and especially FLU with Chk1i or ATRi also resulted in increased levels of ATM and p21. These proteins colocalized with 53BP1 and were distributed throughout the whole nucleus similarly to γH2AX (Fig. 3A,B). The increased expression of p21 was additionally found in MCF7 cells during their exposure to FLU and Chk1i for 48 h as well as in these cells removed from the RS to fresh medium for 24–72 h or 5 days (Fig. 3C).

WSU‐NHL cells were the most sensitive to FLU with negligible additive effect of kinase inhibitors. Pan‐nuclear γH2AX appeared in these cells as soon as 14 h after RS induction by exposure to 5 μg·mL^−1^ of FLU combined with Chk1i (Fig. 2B). The proportion of pan‐nuclear γH2AX fractions reflecting RS was not assessable in this cell line due to the high number of apoptotic cells with similar pan‐nuclear γH2AX staining (Fig. 2B, arrows). WSU‐NHL cells differ from the other cancer cell lines used in this study by displaying an almost zero expression of lamin A/C (Table 1). This essential protein of the INM has been found to be very important for maintaining nuclear stability especially after replication fork stalling 39.

### Impact of replication stress on apoptosis induction and cell cycle profile in individual cell lines

WSU‐NHL cells died very rapidly; low levels of early apoptosis appeared after only 14 h of exposure to just FLU and progressively increased up to 24 h when the late apoptotic cell proportion reached around 80% (Fig. 3D,E). A similar picture was then observed for the combination of FLU with individual kinase inhibitors (Fig. 3D,E). Concerning the cell cycle profile, exposure of WSU‐NHL to FLU resulted in a partial G1 arrest (in line with the WT‐p53 status) and the appearance of a sub‐G1 phase predominantly at 14 h of exposure, indicating the presence of apoptosis. The addition of inhibitors to FLU did not change the cell cycle profile significantly with the exception of creating a more pronounced sub‐G1 phase (Fig. 3F).

The treatment of SU‐DHL‐4 cells with FLU (5 μg·mL^−1^) or FLU combined with Chk1i for 4–48 h did not lead to caspase‐3 activation or any change in the level of anti‐apoptotic Bcl‐2 protein (Fig. 3G1). However, some of the studied exposures induced progressive phosphorylation of H2AX with a maximum level observed 24 h after administration of FLU with Chk1i. Interestingly, this phosphorylation coincided with the hyper‐phosphorylation of the RPA34 protein, confirming the highest level of DNA damage. Caspase 3 activity was also not observed in the cells removed from 48 h of FLU exposure to fresh medium and cultured for an additional 48 h (Fig. 3G2). During this period the level of γH2AX remained high (Fig. 3G2). By contrast, the cells removed from 48 h of exposure to FLU with Chk1i displayed a progressive increase of active caspase‐3 reaching a maximum 48 h after being cultured in new medium (Fig. 3G3).

**Figure 4:**
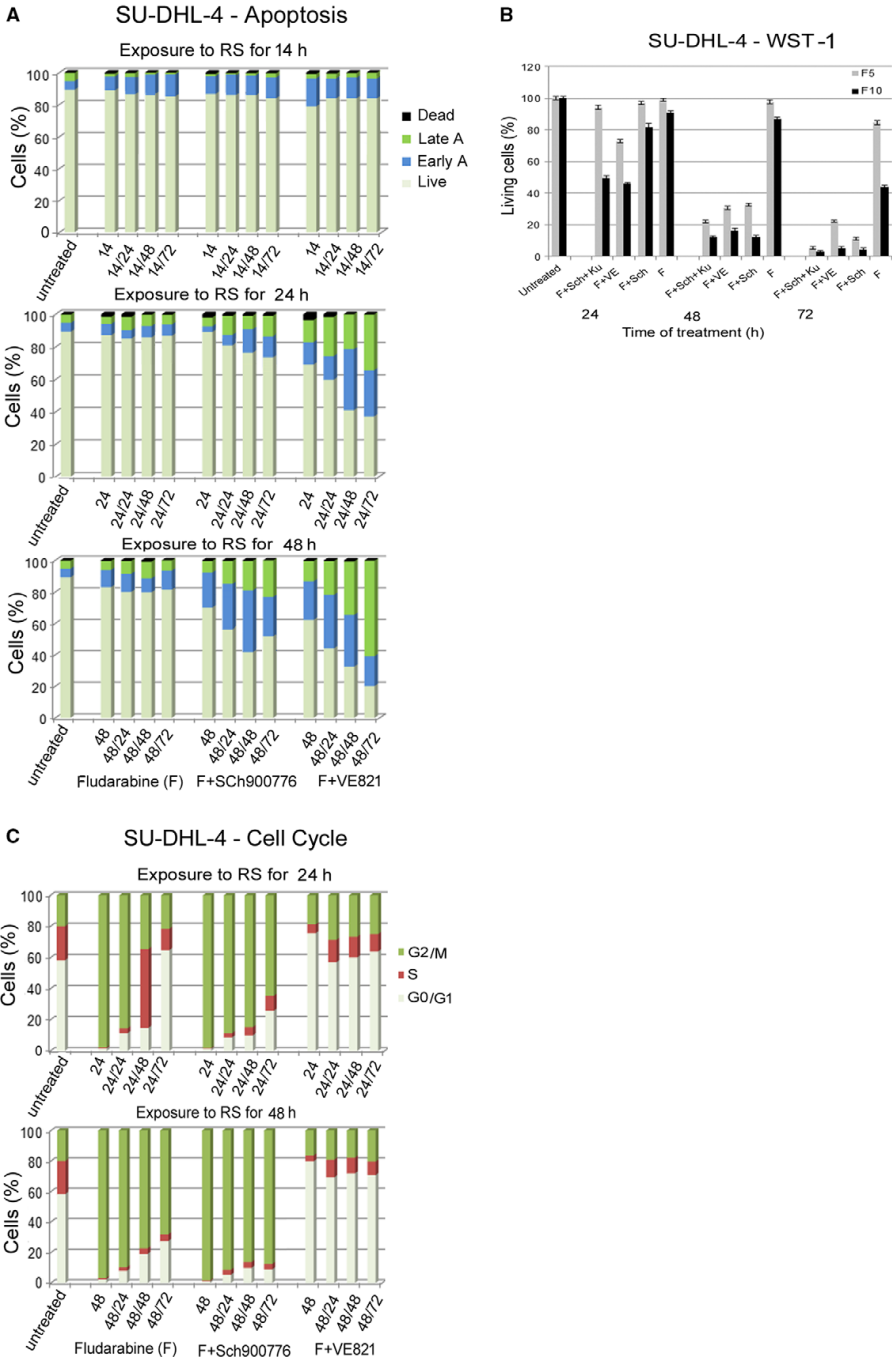
Impact of RS on apoptosis induction and cell cycle profile in SU‐DHL‐4 cells. (A) Proportions of apoptotic cells exposed to FLU 5 μg·mL^−1^ (F), F with Sch900776 (F + Chk1i), and F with VE821 (F + ATRi) for 14, 24 and 48 h and after the release of the cells to fresh medium and additional culture for 24, 48 and 72 h. All results are presented as average from three independent experiments. (B) Measuring of overall viability of cells exposed to FLU (F), F with Chk1i (F + Sch), F with ATRi (F + VE), and F with Chk1i and ATMi (F + Sch + Ku) for 24, 48 and 72 h. The cell viability values detected by metabolic WST‐1 assay are presented as mean of three independent experiments expressed as SEM (each experiment was performed in five different wells seeded with 5 × 10^4^ cells). (C) Distribution of cells in the cell cycle after exposure to FLU (F) alone or FLU with Chk1i (F + Sch900776) or FLU with ATRi (F + VE821) for 24 or 48 h and after release of the cells to fresh medium and additional culture for 24–72 h. All results are presented as average from three independent experiments.

This result was supported by flow cytometry analysis using Annexin V/propidium iodide staining (Fig. 4A). A slightly increased level of early apoptosis was detected in SU‐DHL‐4 cells treated for 48 h with FLU and Chk1i with further increase of early and late apoptosis after the transfer of these cells to fresh media. A similar phenotype during the RS, but with more pronounced apoptosis after the change to new medium, was also observed in the treatment with FLU and ATRi (Fig. 4A). The effect of the inhibitors on apoptosis potentiation (compared to FLU on its own) was also apparent in the metabolic WST‐1 assay measuring overall cell viability (Fig. 4B).

Cell cycle analysis revealed a remarkable phenotype: virtually all SU‐DHL‐4 cells accumulated in G2/M phase after exposure to FLU or FLU with Chk1i for 24 and 48 h (Fig. 4C). The cells removed from 24 h of exposure to FLU recovered from this arrest during a subsequent 72‐h culture in fresh medium and returned to their original cell cycle profile. However, cells exposed to FLU for 48 h and to FLU with Chk1i for 24 h did not recover during this time; about 65% of the cells remained in G2 phase after 72 h in fresh medium and only 5–10% reached S phase, i.e. the subsequent cycle (Fig. 4C). Cells exposed to FLU with Chk1i for 48 h recovered from G2/M arrest with greater difficulty. In striking contrast, the cells exposed to FLU with ATRi for 24 and 48 h did not accumulate in G2 phase (Fig. 4C). These cells remained in G1 and their S phase proportion in fact decreased compared to the untreated control. The transfer of these cells to fresh medium resulted in a slight increase of S and G2 phases.

**Figure 5:**
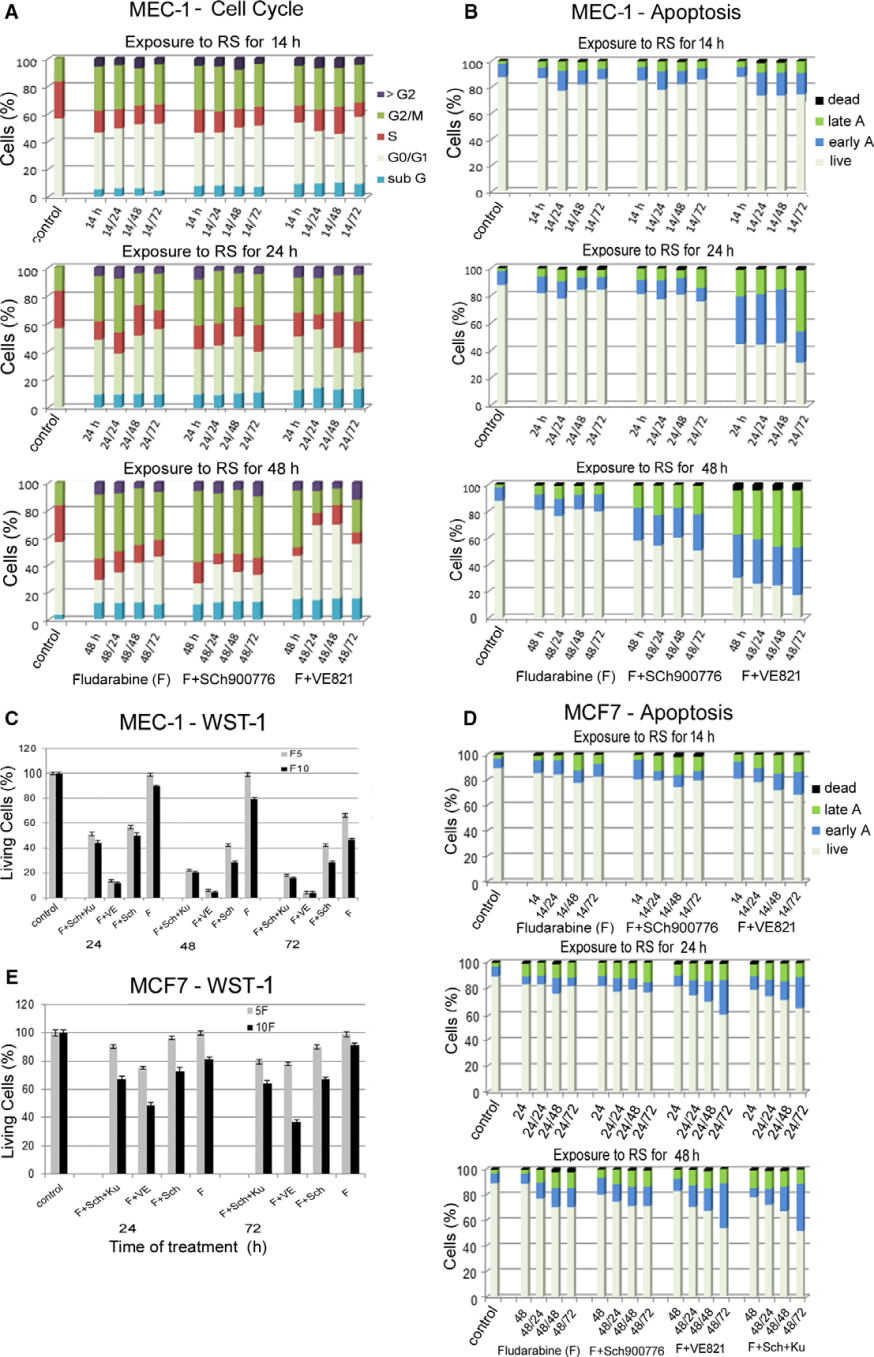
Impact of RS on apoptosis induction and cell cycle profile of MEC‐1 and apoptosis in MCF7 cells. (A) The MEC‐1 cell cycle profile after exposure to FLU 5 μg·mL^−1^ (F), F with Sch900776 (F + Chk1i), and F with VE821 (F + ATRi) for 14, 24, 48 h and after the release to fresh medium and further culture for 24–72 h. All results are presented as average from three independent experiments. (B) Apoptosis in MEC‐1 cells exposed to the same conditions inducing RS as in (A). All results are presented as average from three independent experiments. (C) MEC‐1 cell viability after exposure to FLU (F), F with Chk1i (F + Sch), F with ATRi (F + VE), or F with Chk1i and ATMi (F + Sch + Ku) for 24, 48 and 72 h. The results of metabolic WST‐1 assay are presented as average from three independent experiments, expressed as SEM (each experiment was performed in 5 different wells seeded with 5 × 10^4^ cells). (D) Apoptosis in MCF7 cells exposed to FLU 10 μg·mL^−1^ (F), F + Sch900776, and F + VE821 for 14 h and to FLU10 (F) alone, F + Sch900776, F + VE821, and F + Sch + Ku for 24 and 48 h, and after the release to fresh medium and further culture for 24–72 h. All results are presented as average from three independent experiments. (E) MCF7 cells viability after exposure to FLU10 (F), F with Chk1i (F + Sch), F with ATRi (F + VE), or F with Chk1i and ATMi (F + Sch + Ku) for 24 and 72 h was detected by metabolic WST‐1 assay (each experiment was performed in five different wells seeded with 5 × 10^4^ cells). The results are presented as average from three independent experiments expressed as SEM.

The MEC‐1 cell line has, similarly to SU‐DHL‐4, a mutation in the *TP53* gene, and its exposure to FLU alone or in combination with Chk1i or ATRi resulted in partial G2 accumulation in all three time exposures to RS (Fig. 5A). The transfer to new medium then resulted in distinct outcomes according to the previous RS interval: no significant change in cells exposed to the RS for 14 and 24 h, and partial release from G2 arrest in cells exposed to FLU for 48 h (Fig. 5A). In addition, the cells exposed to FLU and ATRi for 48 h prominently accumulated in G1 phase after incubation in new medium.

Interestingly, MEC‐1 cells also displayed sub‐G1 and post‐G2 phases of the cell cycle which could be caused by aggregates generated from fragmented chromosomes or cell doublets. Chromatin fragments might also come from chromatin released through fissures in inflated nuclei or from disintegrated chromosomes from mitotic catastrophe. There were about 10% of inflated nuclei in the population of MEC‐1 cells exposed to FLU and FLU with Chk1i or ATRi. Frequent fissures were observed in the internal nuclear membrane of such nuclei enabling the release of chromatin into the cytoplasm (Fig. 1C). Many of the MEC‐1 cells treated with FLU, especially in the presence of Chk1i or ATRi, were not able to attach to positively charged slides and dropped out. Unlike MEC1, WSU‐NHL cells also exhibiting a sub‐G1 phase did not drop out from slides and even the apoptotic cells of this cell line attached to microscopic slides; their disintegrated chromatin was clearly distinguished from normal nuclei (Fig. 2B). The distinct behavior of these two cell lines indicates that their sub‐G1 phases could have different origins. In WSU‐NHL cells the fragmented chromatin of the sub‐G1 phase could come from apoptosis but in MEC‐1 cells from mitotic catastrophe and chromatin released through fissures in the nuclear membrane of inflated nuclei.

The results of apoptosis analysis in MEC‐1 cells are summarized in Fig. 5B. FLU on its own had only a negligible impact regardless of the RS exposure length. However, apoptosis was enhanced by the co‐treatment of FLU with Chk1i and especially ATRi. This potentiation of the effect of FLU through the DDR kinase blockade (including ATM) was also apparent in the metabolic WST assay measuring overall cell viability (Fig. 5C). It indicates that impairment of the cell cycle checkpoints reduces the ability of cells to repair induced DNA damage resulting in increased cell death.

Despite the aforementioned γH2AX accumulation in MCF7 cells exposed to RS, these cells showed relatively high resistance to apoptosis (Fig. 5D). The only exception was to exposure to higher FLU concentration (10 μg·mL^−1^) with parallel ATR inhibition (both for 24–72 h) (Fig. 5E).

### Effect of replication stress on the level of lamin B receptor and lamin B1

**Figure 6:**
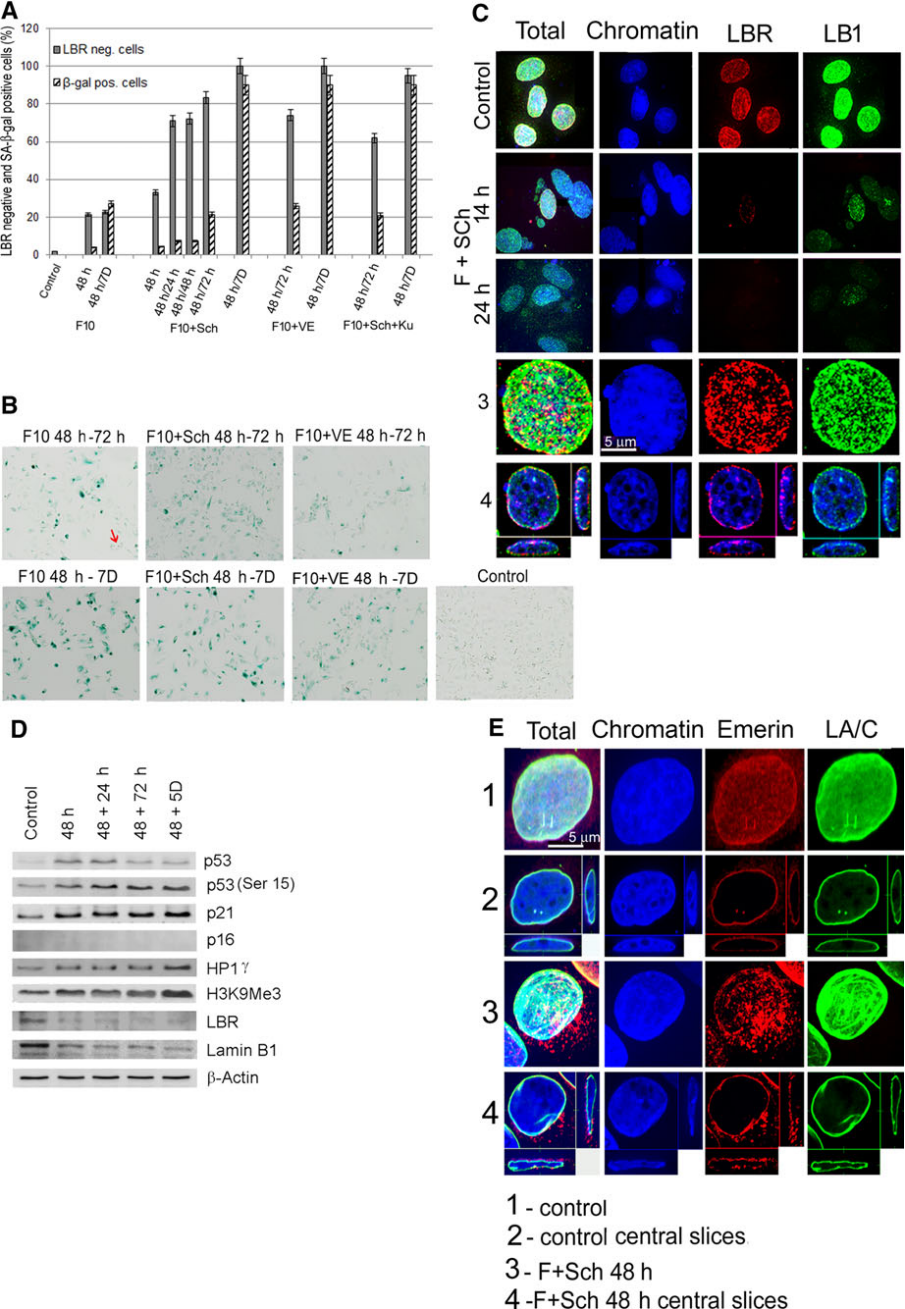
Senescence induced in MCF7 cells after exposure to RS. (A) Proportions of LBR‐deficient and SA‐β‐gal‐positive cells after exposure to FLU 10 μg·mL^−1^ (F10), F10 with Chk1i (F10 + Sch), F10 with ATRi (F10 + VE), and F10 with Chk1i and ATMi (F10 + Sch + Ku). The mean values of LBR‐deficient and SA‐β‐gal‐positive cells were counted from 80 to 100 cells in each of the three repetitions in each sample. The bars represent SEM. (B) Senescent, SA‐β‐gal‐positive cells (blue‐green). Arrow indicates a colony of dividing cells. (C) MCF7 cells displayed in the confocal microscope's field and painted with LB1 (green) and LBR (red) antibodies after exposure to F10 + Sch for 14 and 24 h. Fields 3 and 4 represent a total image and central slice, respectively, through a control cell nucleus containing both LB1 and LBR. (D) Changes in the level of selected proteins associated with senescence phenotype induced by cell exposure to F10 + Sch for 48 h followed by the release of cells to fresh medium and further culture for 24 h, 72 h and 5 days. (E) Lamin A/C and emerin in MCF7 cells after exposure to F10 + Sch for 48 h. While lamin A/C does not change, emerin partially changes its location from nuclear membrane to the cytoplasm. Scale bar: 5 μm.

The exposure of the MCF7 cell line to FLU alone or in combination with Chk1i, ATRi or ATMi led to the transition of the majority of these cells to senescence characterized by the activation of SA‐β‐gal. This phenotype reached a maximum at day 7 of the exposure of the cells to RS (Fig. 6A,B). In addition, cells undergoing senescence reduced the level of LBR and LB1 (Fig. 6C,D). While LBR and LB1 were reduced at the beginning of RS exposure (Fig. 6A,D), the expression of SA‐β‐gal increased slowly; the activity of this enzyme coincided (from the point of view of cell numbers) with the loss of LBR and LB1 up to day 7 of RS exposure (Fig. 6A). Senescent MCF7 cells were also characterized by extensive chromatin condensation as evidenced by the increased level of histone H3K9Me3, phosphorylation of p53 (Ser15) and increased p21 protein level (Fig. 6D). By contrast, the expression of p16 was undetectable in these cells. MCF7 cells also did not change in the expression of polymeric lamin A/C; however, we noted a considerable dispersion of emerin from the nuclear lamina to the cytoplasm after RS exposure (Fig. 6E).

**Figure 7:**
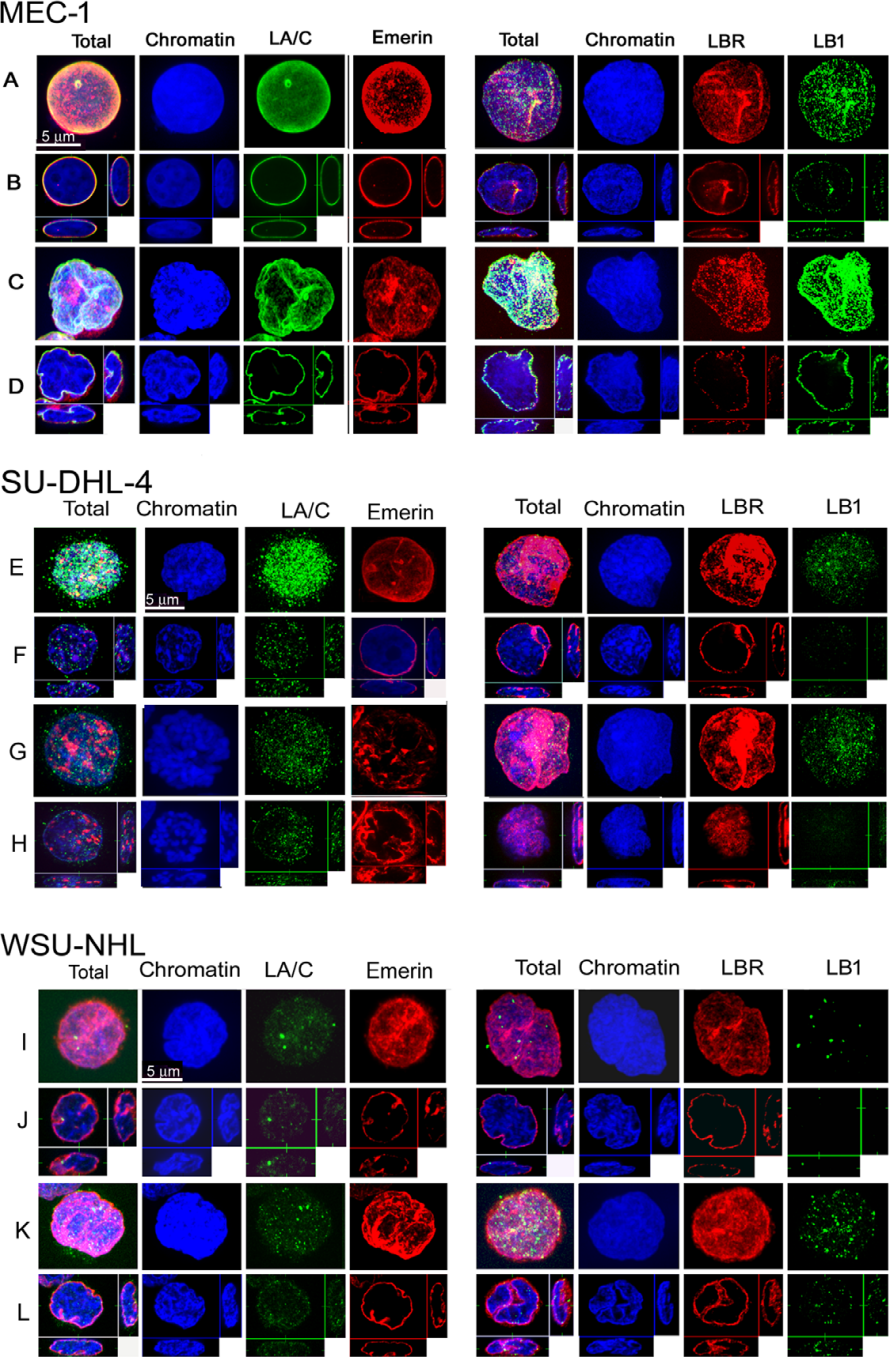
Presence of lamins in lymphoid cell lines MEC‐1, SU‐DHL‐4 and WSU‐NHL. MEC‐1 cells have polymeric LA/C, LB1, LBR and emerin; SU‐DHL‐4 is lacking a confluent lamin A/C layer on the INM; and WSU‐NHL does not express almost any lamin A/C and very low level of LB1. For MEC 1 cells: (A) control cell; (B) central slice through a control cell; (C) cell exposed to FLU 5 μg·mL^−1^ and Chk1i (SCH900776) for 48 h; (D) central slice through the cell exposed as in (C). For SU‐DHL‐4 cells: (E) control cell; (F) central slice through a control cell; (G) cell exposed to FLU 5 μg·mL^−1^ and Chk1i (SCH900776) for 48 h; (H) central slice through the cell exposed as in (G). For WSU‐NHL: (I) control cell; (J) central slice through a control cell; (K) cell exposed to FLU 5 μg·mL^−1^ and Chk1i (SCH900776) for 48 h; (L) central slice through the cell exposed as in (K). Scale bars: 5 μm.

Similarly to MCF7, the MEC‐1 cell line also exhibited polymeric LA/C, LB1, LBR and emerin (Fig. 7A,B, MEC‐1) and the level of LB1 seemed to increase slightly during the exposure to RS (Fig. 7C,D, MEC‐1). Cell lines SU‐DHL‐4 (Fig. 7E,F,G,H, SU‐DHL‐4) expressed a high level of lamin A/C; however, this lamin did not form a confluent layer on the INM of this cell line as in MEC‐1 (Fig. 7A,B) and MCF7 cells (Fig. 6E1,2). Contrary to these cells, WSU‐NHL (Fig. 7I,J, WSU‐NHL) did not express almost any LA/C. Both these cells expressed emerin, LBR and LB1 (Fig. 7E,F, SU‐DHL‐4). The levels of LBR and the very low level of LB1 were not changed by any treatment in the WSU‐NHL cell line (Fig. 7I,J,K,L, WSU‐NHL). The absence of lamin A/C and the low level of LB1 could influence the high sensitivity of these cells to RS resulting in rapid cell death. The enzyme SA‐β‐gal was not expressed in any of the lymphoid cell lines, which indicates that apoptosis, rather than senescence, is a primary mechanism by which these cells responded to the RS.

## Discussion

In this work we investigated the effects of RS on the transition of lymphoid and mammary carcinoma cell lines to apoptosis or senescence. The RS was induced by FLU alone 31 or FLU combined with the selective Chk1 inhibitor SCH900776 32, 33 or the selective ATR inhibitor VE821 16, 34. Chk1 inhibitors represent a promising type of agent that potentiate the cytotoxicity of DNA damaging drugs 40, 41, 42. It is generally accepted that the transformation of cells to senescence after their exposure to DNA damaging agents requires functional p53 19, 20, 21, 22, 23, 24, 43, and therefore we also considered this aspect in our study. WSU‐NHL cells harbored WT‐p53 and were terminated by apoptosis very rapidly after only a short exposure to FLU, with negligible further impact of Chk1 or ATR inhibition (Table 2).

**Table 2:** Response of cell lines to RS. EA, early apoptosis; PT, post‐treatment; total apop., total apoptosis

Cell line properties and reactivity to RS	Cell line			
	WSU‐NHL	SU‐DHL‐4	MEC‐1	MCF7
P53 status	Wild‐type	Mutation	Mutation	Wild‐type
Lamin A/C status	Absence	Mutation	Normal	Normal
Lamin B1 status	Low level	Normal	Normal	Normal
Induction of senescence, LBR and LB1 loss	−	−	−	+
Induction of early and late apoptosis after exposure to Flu and Flu + Chk1i	55% 14 h 90% 24 h	60% 48 h total apop.	45% 24 h total apop.	30% 48 h total apop. 20% EA
Induction of apoptosis after Flu + ATRi	−	48 h total apop.	85% 48 h Total a	45% 24 h total apop. 30% EA
Induction of sub‐G1 phase	6–14 h PT	−	14–48/72 h	−
Induction of post‐G2 phase	−	−	14–48/72 h	−
Chromatin condensation to metaphasic chromosomes	+	+	+	+

The other WT‐p53 cell line, MCF7, was transited to senescence, especially after exposure to FLU with the kinase inhibitors. We found that WSU‐NHL cells contained almost no LA/C and possessed only low levels of LB1, which was in contrast to MCF7. Both these lamins have a primordial role in the attachment of heterochromatin to the INM by means of specific INM proteins to create a functional chromatin structure 44, 45, 46. LBR forms such a tether, or linkage, together with LB1 in proliferating cells (embryonic and undifferentiated) and LEM domain proteins, as well as some not yet fully studied proteins, with LA/C in differentiated cells 46. The absence or low levels of these lamins threaten nuclear integrity, chromatin structure and function 47 and makes cells more susceptible to RS. Singh *et al*. 39 showed that LA/C‐deficient cells are very sensitive to RS. After exposure to hydroxyurea, the cells displayed defective repair protein recruitment, a higher frequency of chromosomal aberrations as well as an impaired replication restart. These findings show that LA/C is required for maintaining genomic stability following replication fork stalling, in order to facilitate DNA damage repair and fork recovery. It is therefore very probable that the absence of LA/C together with a low level of LB1 contributed significantly to the inability of WSU‐NHL cells to repair DNA damage and restart stalled replication forks after exposure to FLU and Chk1i and so led to cell death.

Only a very low fraction of MCF7 mammary carcinoma cells were terminated by apoptosis after exposure to FLU with Chk1i or ATRi, while the majority of these cells exhibited permanent cessation of proliferation and cellular senescence. This cell line responded to DNA damage induced by RS similarly to that induced by γ‐irradiation 25. Based on our recent results, where we showed that the loss of LBR and LB1 in the onset of senescence led to the degradation of constitutive heterochromatin in lamin associated domains, in which chromatin is attached to nuclear membrane, we hypothesize that the cause of this permanent proliferation arrest in senescence could be related to the degradation of constitutive heterochromatin containing lamin associated domains.

SU‐DHL‐4 and MEC‐1 cells have mutated p53, which may compromise cell cycle arrest in the G1/S checkpoint in response to DNA damage, allowing cells with compromised DNA to enter S and G2 phases. In the presence of Chk1i, an additional accumulation of ssDNA could occur making RS more severe and thus leading to cell death 16, 35, 40, 42, 48. However, under some conditions, the combined deficiency of p53 with Chk1 abrogation could lead to mitotic catastrophe 6, 9, 49, 50. The experiments of Wang *et al*. 51, Brough *et al*. 12 and Toledo *et al*. 35 showed that Chk1 inhibitors were particularly toxic for cancer with deficient p53 showing some kind of synthetic lethality. The responses of SU‐DHL‐4 and MEC‐1 to the RS induced by FLU and inhibition of Chk1 were not the same, indicating a difference in the endogenic RS of these cell lines 52, 53. SU‐DHL‐4 cells arrested at G2 phase slowly repaired their damaged DNA and then returned to a new cell cycle in accordance with the RS extent; those cells that did not manage to repair DNA in time were terminated by apoptosis. In contrast to the effect of FLU with Chk1i, FLU with ATRi killed almost 80% cells in SU‐DHL‐4 and MEC‐1 cell lines treated for 48 h. The increased sensitivity of the cells to ATRi compared to Chk1i reflects the central role of ATR as an essential regulator of genome integrity 2, 4. ATR activates Chk1 in S and G2 phases, and if its function is abrogated (e.g. by an inhibitor), the Chk1 and other ATR substrates are deactivated 2, 4, dormant origins fire and ssDNA progressively depletes all nuclear RPA, ultimately resulting in cell death 52, 53. However, recent results show 54 that if ATR is abrogated, Chk1 is activated by DNA‐PK. This Chk1 backup activity suppresses origin firing, reduces ssDNA and allows cells to recover from ATR inhibition. Buisson *et al*. 54 also observed that prolonged ATR inhibition increased cell death, which is in accordance with our results.

Unlike in SU‐DHL‐4, MEC‐1 cells exhibited a low expression of Chk1 48 and normal levels of LA/C and LB1. This low expression of Chk1 could reduce the activity of both checkpoints (G1/S and G2/M) probably rendering these cells more susceptible to the RS. This would result in a decreased capacity to repair DNA damage during the S‐phase and the G2 phase, and the transition of cells with under‐replicated DNA to mitosis leading to mitotic catastrophe accompanied by chromatin fragmentation. The presence of a sub‐G1 peak and a post‐G2 peak after only a short exposure to FLU and increasing with time of cell exposure, especially if accompanied by Chk1 or ATR inhibition, could be related to the reduced expression of Chk1 in MEC‐1 cells. Our results support the observation of an extensive chromatin fragmentation in MEC‐1 cells by Zemanova *et al*. 48 after treatment with FLU and Chk1i. Toledo *et al*. 52, 53 showed that the response to checkpoint inhibitors depends on tumor specific conditions such as endogenous RS that could influence the rapidity of RPA exhaustion protecting ssDNA from breakage resulting in replication collapse and cell death.

Cells of all four cell lines exhibited widespread H2AX phosphorylation (γH2AX) and unusual chromatin condensation after exposure to FLU alone or FLU with Chk1i or ATRi. The chromatin structure of cancer cells is commonly more condensed compared to normal cells but the reason is not quite clear; it is probably related to genetic changes that are responsible for tumor transformation. In numerous nuclei exposed to RS, part of the chromatin was condensed to metaphasic chromosomes with two chromatids joined in the centromere that were well distinguished after the immunodetection of γH2AX and 53BP1, since both these antibodies bind to these chromosomes. However, only a part of the chromatin reached this high degree of condensation in a nucleus, while the remainder presented a lower degree of condensation. We hypothesize that metaphasic chromosomes immunodetected by antibodies to γH2AX and 53BP1 contained high levels of ssDNA surrounding a dense network of stalled replication foci in the cells arrested in the S or G2 phase. This damage could promote chromatin condensation in these regions, since reports have shown that fork stalling predominantly leads to heterochromatinization 55. Although the mechanism linking RS with heterochromatin formation remains largely unknown, it has been reported that some proteins participating in chromatin condensation as cohesins accumulated at replication sites when DNA synthesis was interrupted 56. Their presence was found to be vital for the recovery of stalled forks in budding yeast and human cells where they also interacted with Sir2 histone deacetylase 57. An important role in the formation of this silent heterochromatin triggered in mammalian cells by RS is the phosphorylation of the histone variant H2AX 1, 6, 40, 58. This histone modification supports chromatin remodeling and the recruitment of repair proteins 58. In addition to γH2AX, it has recently been shown that 53BP1 forms large nuclear structures around persisting DNA lesions induced by RS to protect these vulnerable regions until repair 3, 11. Both γH2AX and 53BP1 were frequently bound to whole condensed chromosomes or only to a part of these chromosomes in cells exposed to replication inhibitor FLU and its combination with Chk1i. Understanding of the conformations adopted by long stretches of ssDNA has been hindered by a lack of defined substrates greater than 150 nucleotides (nt), and the absence of high‐resolution approaches. A recently published study 59 has detailed the generation of long ssDNA containing distinct repeating nucleotide sequences and provided new information about the architecture of replication forks. The authors discovered several kinds of macroscopic folding that ssDNA segments from 200 to 2000 nt or more in length can spontaneously adopt at sites of transcription, recombination and uncoupled replication forks. If the ssDNA is long enough and consists of arrays of nucleotide repeats, it is possible for it to create such structures. On the basis of these results we can assume that this folding could occur even in cells exposed to RS that have not yet reached the prometaphase. Chromatin of these cells does not have a unified structure because the content, quality and sites of ssDNA in individual chromosomes differ. The cells with this unusual structure are probably arrested in the S or G2 phase.

In summary, our results show that FLU combined with Chk1i and especially ATRi was efficient in the elimination of mammary carcinoma and lymphoid cancer cells. While mammary carcinoma cells went to senescence, WSU‐NHL cells, which lack lamin A/C, died rapidly by apoptosis. The other lymphoid cancer cell lines (SU‐DHL‐4 and MEC‐1) terminated at different rates after treatment, indicating the presence of distinct internal RS, influencing the sensitivity of cells to the stressors used in this work.

## Conclusions

Our results show that RS induced by FLU and reinforced by a parallel inhibition of kinases responding to DNA damage may lead to diametrically distinct responses in individual cancer cell types. This diversity occurs despite the similar impact of the RS on chromatin structure and the DNA damage accumulation accompanied by widespread γH2AX. We observed that individual cancer cell lines do not cope with RS in the same manner. Thus, the mammary carcinoma cells possessing WT‐p53 and a normal level of all lamins underwent a transition to senescence accompanied by a significant reduction of LBR and LB1 in response to the RS. By contrast, lymphoid cancer cells possessing WT‐p53 in the absence of lamin A/C together with low levels of LB1 responded to the same stimuli by rapid death by apoptosis. Lymphoid cells with the mutation status of p53 and normal or mutated lamin A/C responded to RS according to their endogenous RS determined by tumor‐specific conditions. It is known that LA/C is required for maintaining genome stability following replication fork stalling; however, how lamin deficiency influences the cell decision to terminate by apoptosis or survive at permanent arrest of proliferation after exposure to RS has still to be studied.

## Conflict of interest

The authors declare no conflict of interest.

## Author contributions

EL conceived the study, designed and performed most experiments and wrote the manuscript; AB and LŠ performed western blotting, WST assays and data analysis; MŘ and JV performed flow cytometric analysis and data analysis; SK coordinated and supervised the project.
